# Survey of Attitudes toward Uterus Transplantation among Japanese Women of Reproductive Age: A Cross-Sectional Study

**DOI:** 10.1371/journal.pone.0156179

**Published:** 2016-05-20

**Authors:** Iori Kisu, Kouji Banno, Etsuko Soeda, Yuki Kurihara, Miho Okushima, Ami Yamaguchi, Eriko Nakagawa, Kiyoko Umene, Daisuke Aoki

**Affiliations:** 1 Department of Obstetrics and Gynecology, Keio University, School of Medicine, Tokyo, Japan; 2 Faculty of Nursing and Medical Care, Keio University, Tokyo, Japan; 3 Department of Nursing, Keio University Hospital, Tokyo, Japan; VU University Medical Center, NETHERLANDS

## Abstract

**Objective:**

Uterus transplantation (UTx) is a potential option for women with uterine factor infertility to have a child, but there has been no large-scale survey of the views on UTx in women of reproductive age in Japan. The present study was aimed to clarify the views of Japanese women of reproductive age on UTx for uterine factor infertility.

**Methods:**

A questionnaire on UTx was conducted by an Internet research company in December 2014 as a cross-sectional study in 3,892 randomly chosen women aged 25 to 39 years old. Responses were analyzed from 3,098 subjects (mean age 32.1±4.2 years old), after exclusion of inappropriate respondents in screening.

**Results:**

Of the respondents, 62.1%, 34.7% and 18.1% favored adoption, UTx and gestational surrogacy, respectively. In contrast, 7.0%, 21.9% and 63.3% opposed adoption, UTx and gestational surrogacy, respectively. In choices of candidates for UTx based on highest priority, deceased persons (33.8%) and mothers (19.0%) were favored as donors, and women with congenital absence of the uterus (54.4%) and hysterectomy due to a malignant uterine tumor (20.0%) as recipients. Regarding societal acceptance of UTx, the answer rates were 15.7% for "UTx should be permitted", 77.6% for "UTx should be permitted with discussion", and 6.7% for "UTx should not be permitted, even with discussion". Regarding personal opinions on UTx, 44.2% were in favor, 47.5% had no opinion, and 8.3% were against.

**Conclusion:**

Our results suggest that many Japanese women of reproductive age feel that UTx is socially and individually acceptable, but that concerns requiring further discussion remain among these women. There was also a tendency for UTx to be viewed more favorably than gestational surrogacy.

## Introduction

Uterus transplantation (UTx) is a potential option for women with uterine factor infertility (UFI) to have a child [1,2]. After accumulation of data from animal studies, including in primates [3], Brännström et al. achieved the first human delivery after UTx in 2014 [4]. This outcome attracted much attention worldwide and provided considerable hope to women with UFI. In Japan, based on this study, UTx has begun to be recognized as a new assisted reproductive technology (ART) and transplantation technique.

About 3.7% of newborns are born in Japan by ART, including *in vitro* fertilization and embryo transfer (IVF-ET) [5], and this technique has helped many infertile couples. However, women with uterine infertility due to hysterectomy for a malignant uterine tumor, benign disease or postpartum hemorrhage and those with a congenital defect such as that in Mayer-Rokitansky-Küster-Hauser syndrome currently can only have children through adoption or gestational surrogacy. Gestational surrogacy is also restricted due to legal, ethical and religious issues in many countries [6–9], while adoption is uncommon in Japan compared with Europe and the US [10]. In Japan, no legal system has been established for regulating ART, which has been controlled by only guidelines of the Japan Society of Obstetrics and Gynecology [9]. The guideline for surrogacy states that ‘surrogacy is not acceptable’ and that ‘conduct of, involvement in, and mediation of surrogacy should not be allowed’ for the following 4 main reasons: Priority should be given to the welfare of the children to be born; surrogacy is accompanied by physical risks and mental burdens; complex family relationships are produced; and a surrogacy contract is not ethically accepted by many people in society. However, couples are increasingly going to countries such as India, Thailand and Malaysia because gestational surrogacy is not available in Japan, and this is resulting in social problems due to commercial deals of procurers [11].

We estimate that there are 60,000 to 70,000 women of reproductive age with UFI in Japan, which is calculated by the Vital Statistics of Japan and registration of patients of the Japan Society of Obstetrics and Gynecology, and UTx may be a new technique that provides hope to these women. UTx is still in the clinical study stage and the safety and efficacy remain unclear despite several clinical applications [4,12–15], but a Swedish research group has accomplished delivery after UTx and women with UFI have had a child due to UTx [4]. UTx also has ethical problems that must be resolved before wider clinical application [3]. UTx differs from gestational surrogacy and adoption, and its acceptance may depend on the social and religious background in a particular country. Attitudes toward UTx in Japan are unclear. Therefore, it is important to obtain the views of patients and the public in considering clinical use of UTx to prevent the technique being introduced too early and to understand opinions and social needs. The attitude toward UTx may also depend on the age of women of reproductive age. Thus, we conducted a questionnaire survey via the Internet to investigate opinions on UTx of women of reproductive age in Japan and compare the attitude by age as the primary endpoint.

## Materials and Methods

### Internet questionnaire

This study was conducted by an Internet research company that recruited respondents for the questionnaire using media including websites, mail magazines, newspapers and news magazines. Approximately 1.2 million people in Japan of different ages, occupations and residential areas were enrolled as respondents. Respondents obtain points after answering the questionnaire and the accumulated points are converted into products, cash or points for partner companies, as an incentive system. Answers by mobile phone were excluded. Respondents answering by personal computer were included in the analysis. The questionnaire was administered in December 2014. The study was conducted with the approval of the ethics committee of Keio University School of Medicine (No. 20130257).

### Subjects and sampling

The subjects were women aged 25 to 39 years old who were randomly selected from persons registered by the Internet research company. To reduce inappropriate answers and ensure the quality of data, subjects with brief response times in the upper 3% of respondents were excluded. Furthermore, based on the screening system of the Internet research company, respondents who were determined to have given inappropriate answers were excluded as needed and alternative persons were added. After providing information on UTx (Appendix B in S2 Appendix), three questions (Q5-7 in Appendix A of S1 Appendix, questions shown in Appendix B in S2 Appendix,) were asked to confirm that the respondents understood this information. Those who gave a wrong answer were excluded from the study. The subjects were classified into three 5-year age groups (25–29, 30–34, 35–39) and the target number of respondents was set at 1000 in each group (3000 persons in total). Based on anticipated exclusion of respondents due to a wrong answer, the target number was increased by 20% (i.e., 600 more persons) and further increased by 6% (3% for respondents with inappropriate responses, 3% for respondents with brief responses). Finally, data were to be collected from 3816 eligible responders. The respondents were collected based on matching of the percentages of unmarried women and type of residential area (urban and rural, based on prefectures) with these percentages in the Japanese population census. Prefectures were classified as urban or rural, with an urban area defined to be a prefecture with >5 million inhabitants.

### Questions

Questions were developed on the basis of literature on UTx, discussion among experts, media attention, international status of UTx implementation, and patient opinions. The contents of the questionnaire on UTx are shown in Appendix A in S1 Appendix. The survey consisted of 22 questions and was required to be completed within about 15 minutes to prevent respondents giving long answers and to collect appropriate answers. The following questions were included: number of children and history of examination or treatment of infertility (Q1,2), prior awareness of UTx (Q3,4), confirmation of understanding of UTx after receiving an objective explanation (Q5-7), choice of donors and recipients (Q8-11), probable thoughts on UTx with the assumption that the respondent had UFI (Q12-16), comparison with surrogacy and adoption (Q17-20), and social acceptance and personal opinion (Q21-22).

In the explanation of UTx, information was provided as objectively as possible to prevent information bias. Positive and negative aspects of UTx were indicated briefly, given the effort required of respondents to read a long explanation and retain information. The positive aspects were defined as carrying a child in a woman's own uterus and self delivery, and the negative aspects as the need for transplant and the risks for transplant rejection and use of immunosuppressants, as described in Appendix B in S2 Appendix. Choices of donors and recipients (Q8-11) and reasons for a particular view on UTx (Q13-16) were randomized to avoid order bias.

### Statistical analysis

Categorical variables were compared by chi-square test. Comparisons of non-parametric data between two groups and among three or more groups were performed by Mann-Whitney U test and Kruskal-Wallis test, respectively, with a Bonferroni correction for multiple comparisons. Regression analysis for categorical data was conducted to evaluate relationships among variables. Data were analyzed with SPSS version 22. *P* < 0.05 was considered significant and adjusted *P* value was adapted in multiple comparison procedure. Comparison by age (5 years) was assessed as the primary endpoint. The effects of marriage, children, residential data and history of examination or treatment of infertility on answers were evaluated as secondary endpoints.

## Results

### Characteristics of subjects

**Fig 1:**
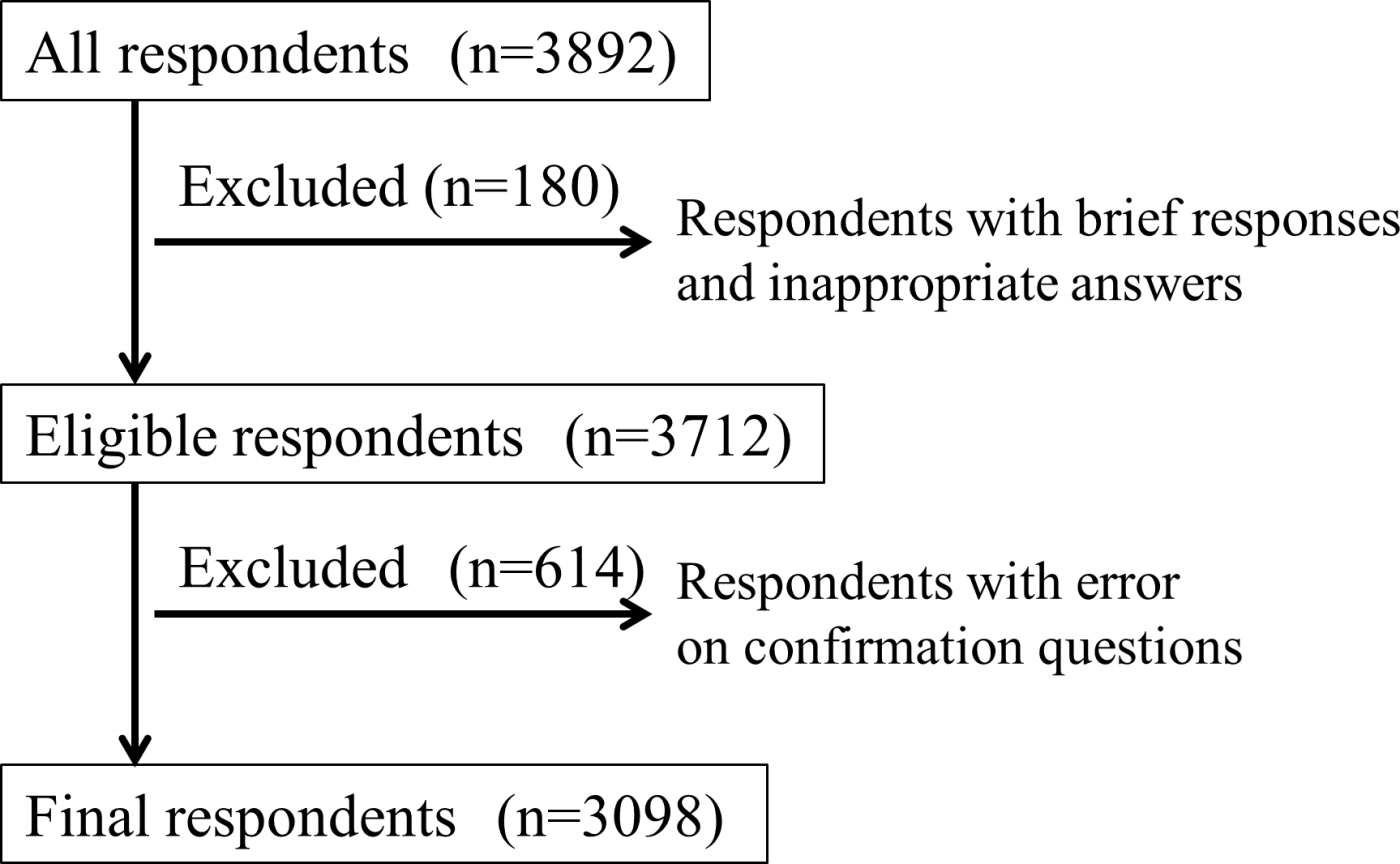
Screening of respondents to the questionnaire.

In this survey, answers were collected from 3892 respondents. A total of 3712 respondents were considered eligible after exclusion of the upper 3% each of inappropriate and brief respondents in screening by the Internet research company. A total of 614 respondents gave the wrong answer to one of 3 questions used to confirm their understanding of UTx, which was almost equal to the expected number with a wrong answer (600 persons). After exclusion of these respondents, data from 3098 eligible subjects were included in the analysis (Fig 1).

The characteristics of the 3098 respondents (mean age: 32.1±4.2 years) are shown in Table 1. The percentages of unmarried women and residential areas per age group corresponded closely to those in the Japanese population census. Of the respondents, 58.9% were women without a child, 14.4% had a history of examination or treatment for infertility (Q1,2), and 17.6% were aware that UTx was used overseas (Q3). These percentages were similar in each age group (p = 0.21). Interest in UTx was indicated by 35.9% of the respondents (Q4), with subjects in the 35–39 age group showing significantly less interest in UTx compared with those in the other two age groups (both adjusted p<0.001).

**Table 1:** Characteristics of respondents.

		Total		25–29		30–34		35–39	
		(n = 3098)		(n = 1042)		(n = 1043)		(n = 1013)	
Married									
	Yes	1904	(61.5%)	428	(41.1%)	692	(66.3%)	784	(77.4%)
	No.	1194	(38.5%)	614	(58.9%)	351	(33.7%)	229	(22.6%)
Residence									
	Urban	1616	(52.2%)	543	(52.1%)	551	(52.8%)	522	(51.5%)
	Rural	1482	(47.8%)	499	(47.9%)	492	(47.2%)	491	(48.5%)
Number of children									
	0	1824	(58.9%)	810	(77.7%)	572	(54.8%)	442	(43.6%)
	1	592	(19.1%)	152	(14.6%)	230	(22.1%)	210	(20.7%)
	2	543	(17.5%)	72	(6.9%)	184	(17.6%)	287	(28.3%)
	≥3	139	(4.5%)	8	(0.8%)	57	(5.5%)	74	(7.3%)
Attitude toward having children in respondents (n = 1824) who do not have a child									
	Want children now	348	(11.2%)	98	(9.4%)	142	(13.6%)	108	(10.1%)
	Want children in the future	893	(28.8%)	491	(47.1%)	259	(24.8%)	143	(14.1%)
	Do not want children	466	(15.0%)	169	(16.2%)	129	(12.4%)	168	(16.6%)
	Currently pregnant	117	(3.8%)	52	(5.0%)	42	(4.0%)	23	(2.3%)
Previous infertility treatment									
Yes	(more than one answer allowed)	445	(14.4%)	75	(7.2%)	168	(16.1%)	202	(19.9%)
	Examination only	333		56		126		151	
	Timing method	364		57		141		166	
	Artificial insemination	133		11		48		74	
	IVF treatment	109		9		36		64	
	Others	10		3		3		4	
No.		2653	(85.6%)	967	(92.8%)	875	(83.9%)	811	(80.1%)
Have you heard that UTx was recently performed in Sweden and Turkey?									
	Yes	545	(17.6%)	168	(16.1%)	199	(19.1%)	178	(17.6%)
	No.	2553	(82.4%)	874	(83.9%)	844	(80.9%)	835	(82.4%)
What is your response when you hear the term 'UTx'?									
	Very interested	66	(2.1%)	17	(1.6%)	30	(2.9%)	19	(1.9%)
	Interested	1048	(33.8%)	363	(34.8%)	390	(37.4%)	295	(29.1%)
	Less interested	1055	(34.1%)	364	(34.9%)	346	(33.2%)	345	(34.1%)
	Not interested	398	(12.8%)	108	(10.4%)	121	(11.6%)	169	(16.7%)
	Not certain	531	(18.2%)	190	(18.2%)	156	(15.0%)	185	(18.3%)

### Choices of candidate donors and recipients

**Fig 2:**
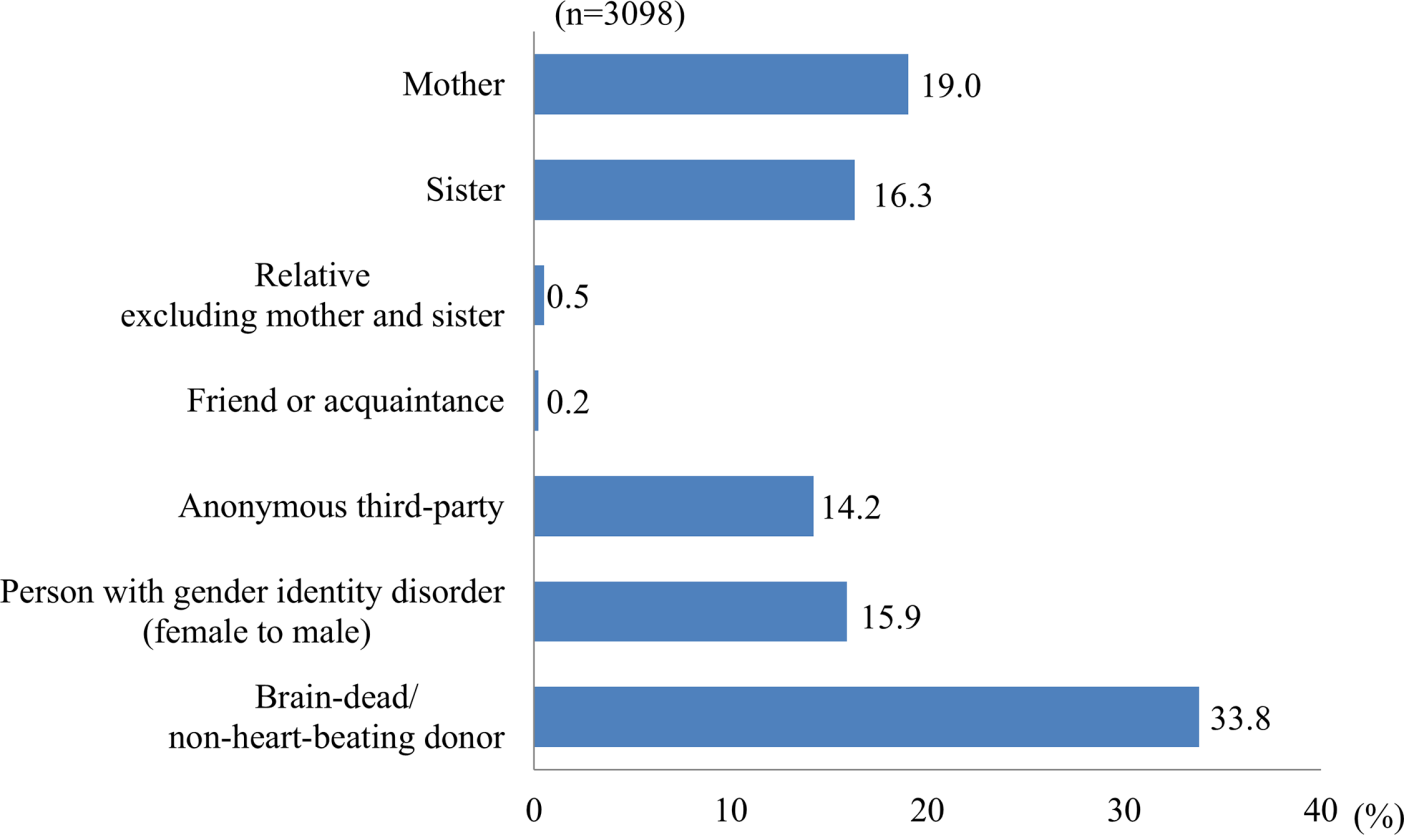
Candidate donors for UTx.

Appropriate donors for UTx (Q8, multiple answers allowed) were considered to be brain-dead/non-heart-beating donors (59.4% of respondents), sisters (42.8%), persons with gender identity disorder (i.e., female to male) (42.3%), mothers (41.9%), and anonymous third-party donors (37.9%). In ranking of these donors (Q9), brain-dead/non-heart-beating donors were ranked first (33.8%), followed by mothers (19.0%), sisters (16.3%), relatives excluding mother or sister (0.5%), and acquaintances (0.2%) (Fig 2). The distributions of answers differed significantly between the 25–29 and 35–39 age groups (adjusted p = 0.034). Sisters were favored as donors significantly more frequently in the 35–39 age group compared to the 25–29 age group (p = 0.009).

**Fig 3:**
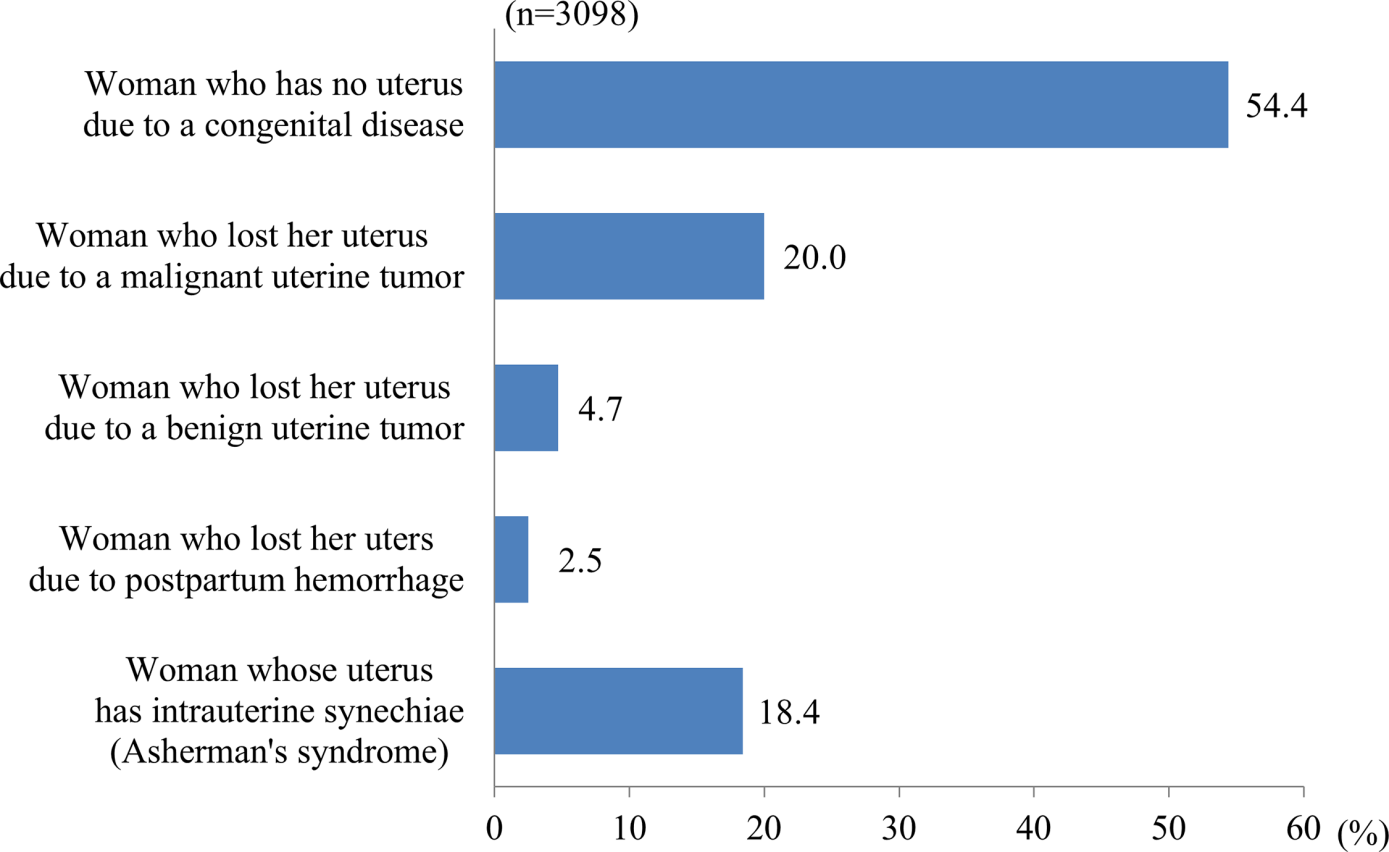
Candidate recipients for UTx.

Appropriate reasons to be a UTx recipient (Q10, multiple answers allowed) were considered to be uterine agenesis (congenital absence of the uterus) (82.5%), hysterectomy due to a malignant uterine tumor (80.6%) or a benign uterine tumor (75.6%), uterine malformation or intrauterine synechiae (78.9%), and hysterectomy due to postpartum hemorrhage (56.8%). In ranking of these conditions (Q11), uterine agenesis was ranked as the highest priority (54.4%), followed by hysterectomy due to a malignant uterine tumor (20.0%) (Fig 3). There was no effect of children on the distribution of answers (p = 0.073). However, marriage significantly influenced the distribution (p = 0.006), with a significantly higher choice of uterine agenesis as a reason for UTx among married women (p = 0.006).

### Thoughts on UTx if the Respondent Imagined having UFI

Regarding the desire for UTx with the assumption that the respondent herself had UFI (Q12), the most common was "No opinion" (53.6%), followed by "I would want UTx" (26.4%) and "I would not want UTx" (19.8%). Of the respondents (n = 817) who did not want UTx, 55.9% (n = 307) answered that they did not want a child in Q1. The rate for not wanting UTx was significantly higher in the 35–39 age group (37.1%, n = 303) than in the other groups (p = 0.006). In regression analysis using age, marriage, children, residential area, and history of examination or treatment of infertility as variables, there were relationships of a desire for UTx with age, children and a history of examination or treatment. Women in the young age group with one or more children and a history of examination or treatment of infertility wanted UTx at a significantly higher rate (p<0.001).

**Fig 4:**
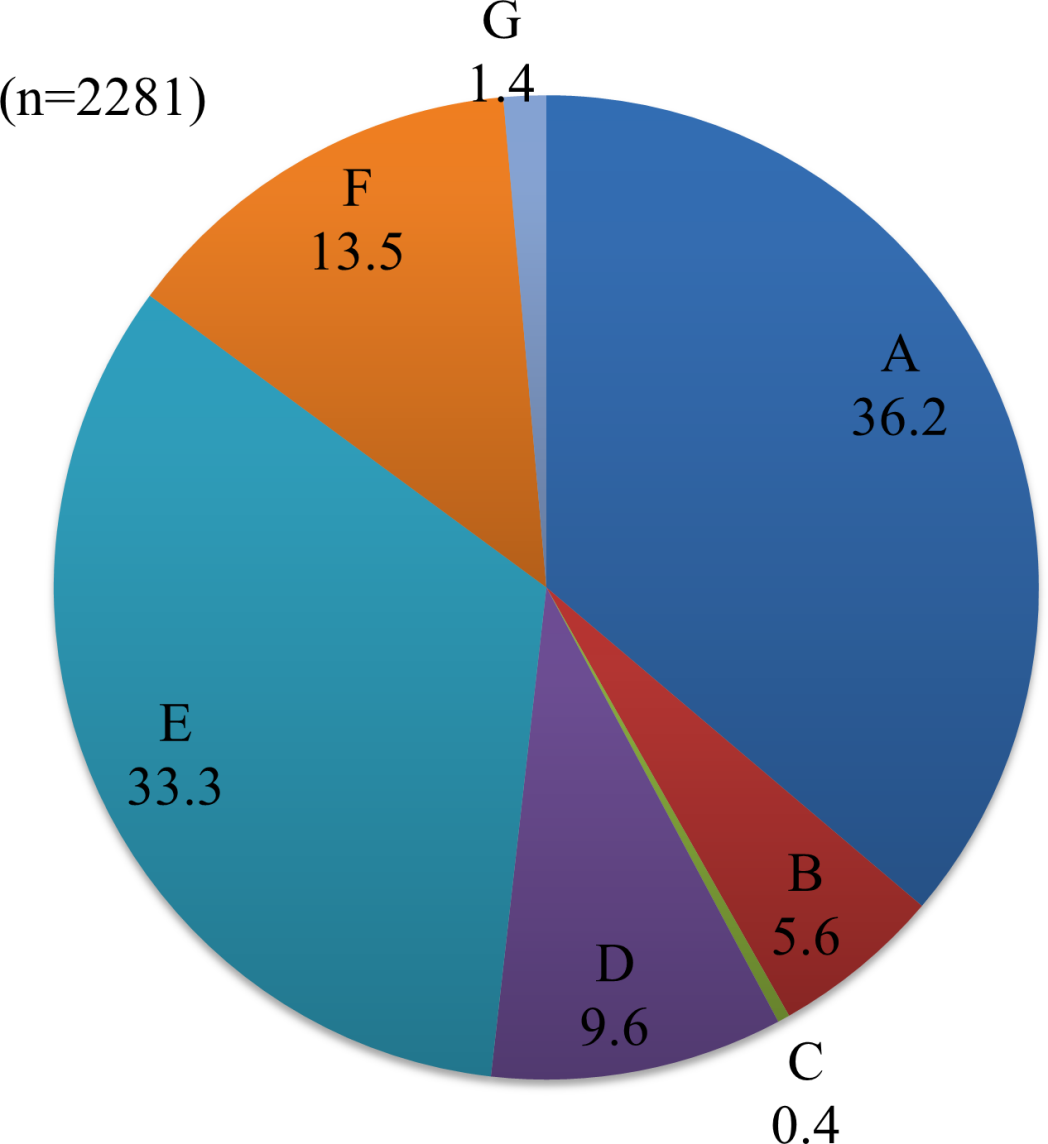
Reasons for wanting UTx. A. We want a child with genes inherited from us. B. There are no ethical problems because the sperm and ovum are ours, even though the uterus is not. C. If the uterus is eliminated after delivery, immunosuppressants are transiently administered. D. Pregnancy and delivery improve maternal feelings, in contrast to gestational surrogacy and adoption. E. Women suffering from uterine factor infertility will have the hope to have a child. F. We want a child by self-delivery who is recognized as ours (the couple's) in the Japanese Civil Code. G. Other reasons.

**Fig 5:**
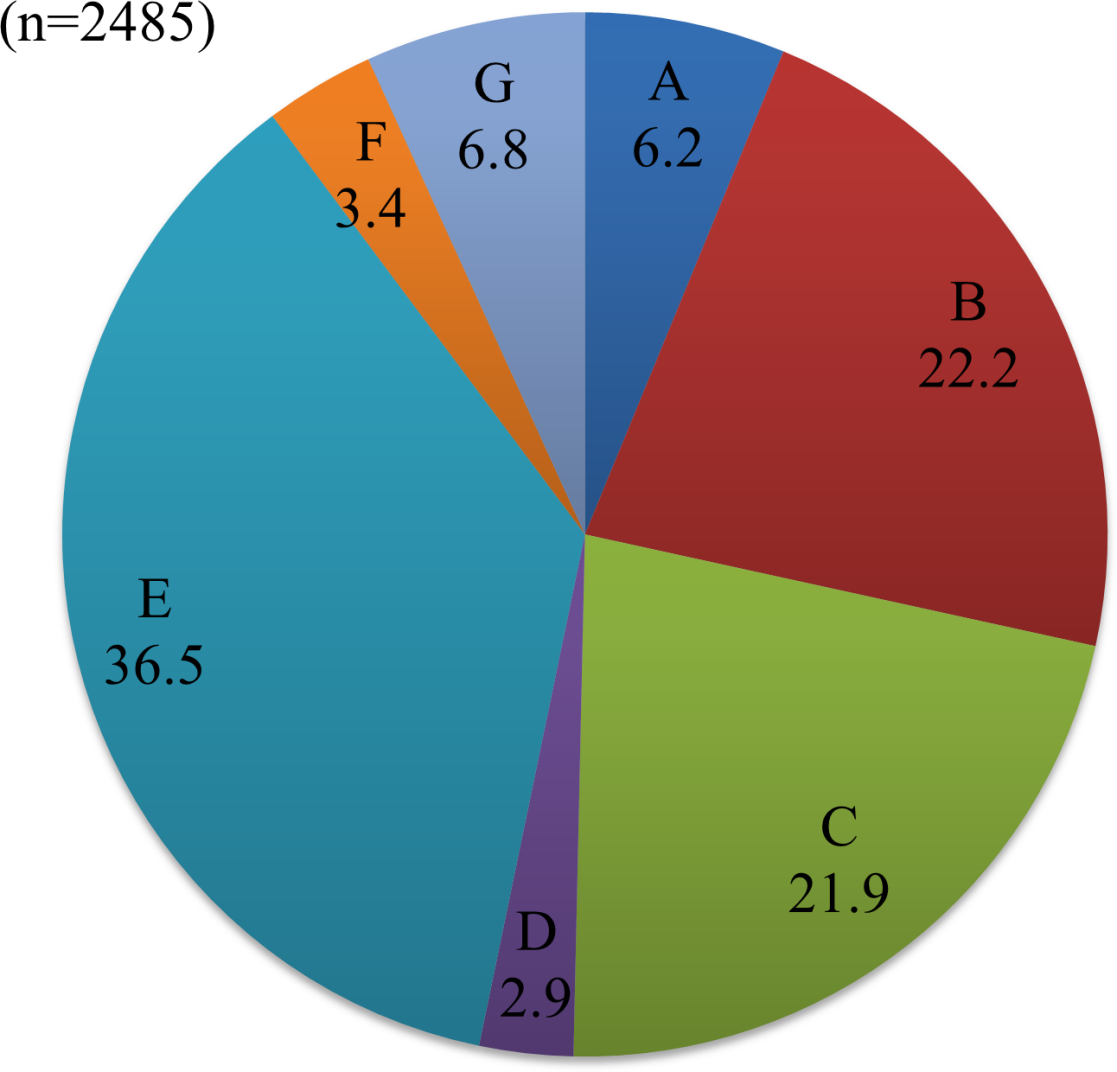
Reasons for not wanting UTx. A. Transplant should be performed only for vital organs, and not for having a child. B. I am afraid of the surgery itself. C. I am worried about the effect of immunosuppressants on a child (anomaly). D. Immunosuppressants are needed. E. I do not want pregnancy and delivery to be risks for myself and the donor. F. Hope should not be easily given to women who have lost their uterus. G. Other reasons.

Reasons for UTx to be available (Q14, n = 2281) are shown in Fig 4. Among respondents who gave the most common answer "Wanting a child with genes inherited from us" (n = 825, 36.2%), there was a high percentage of married women with one or more children (n = 378, 45.8%) and this rate was significantly higher than that in the total population (39.4%) (p = 0.001). The main reasons given for UTx not to be available (Q16, n = 2485) are shown in Fig 5. Of subjects who were married but did not want a child (n = 122), 50.0% (n = 61) chose "I don't want pregnancy and delivery to place myself and the donor at risk", and this rate was significantly higher than that in the total population (36.5%) (p = 0.003).

### Comparison with surrogacy and adoption

**Fig 6:**
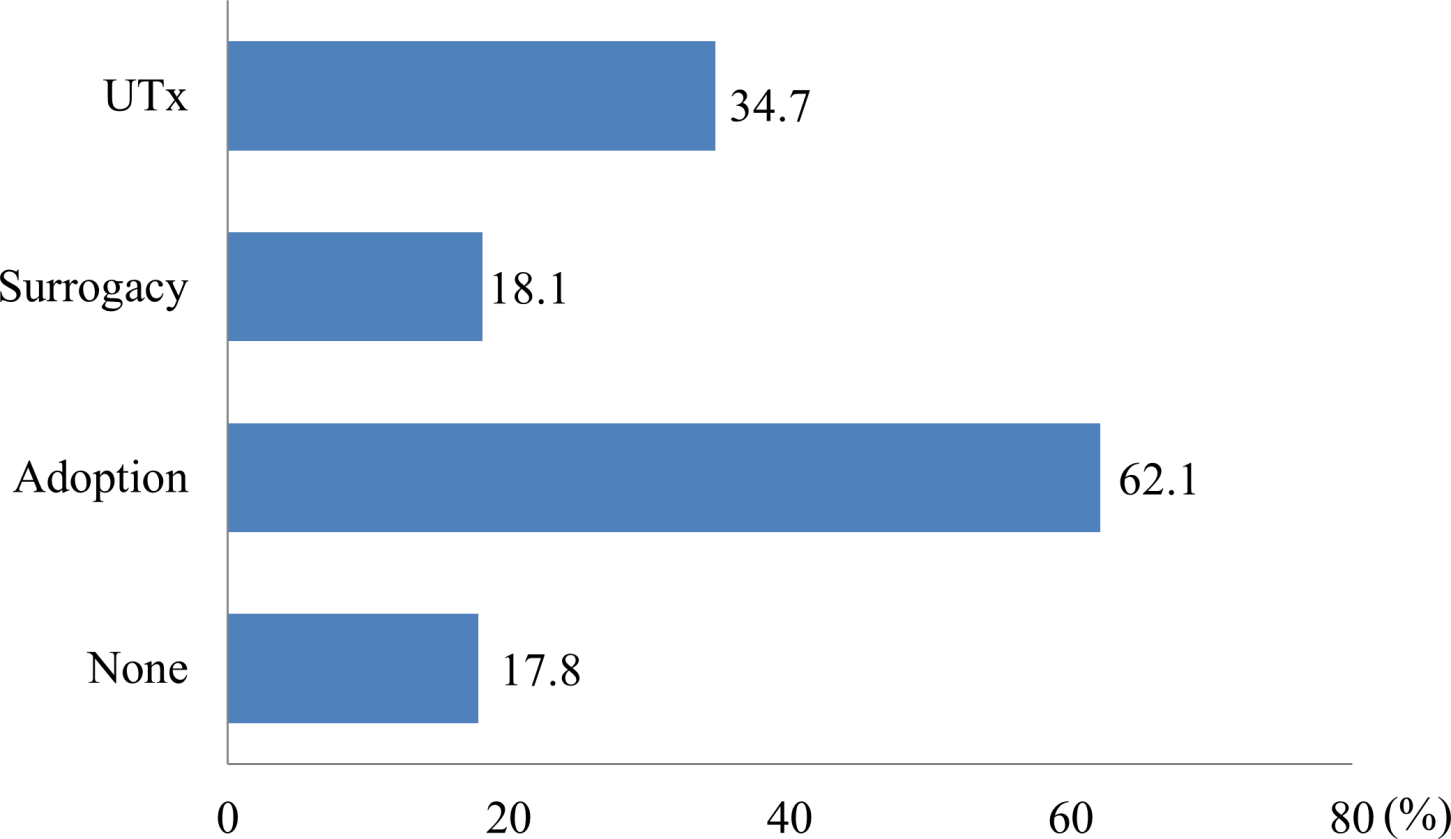
Support for treatment options for uterine factor infertility. (Multiple answers allowed).

Support for treatment options for uterine factor infertility (Q17, multiple answers allowed) are shown in Fig 6. For the top choice for these options (Q18), the rates were 29.6%, 8.0%, and 62.4% for UTx, gestational surrogacy, and adoption, respectively.

There were no effects of age group and residential area on choice of option (p = 0.897 and 0.284, respectively). The percentages of married women who chose UTx, gestational surrogacy and adoption were 65.3%, 61.6% and 58.8%, respectively, with UTx favored significantly by married women, compared to adoption (p = 0.003). Similarly, the percentages of women with one or more children who chose UTx, gestational surrogacy and adoption were 46.9%, 40.0% and 38.5%, respectively. UTx was chosen significantly by women with children more than adoption (p<0.001). Among women who thought they originally did not want a child (n = 361) the percentages that chose UTx, gestational surrogacy and adoption were 15.2% (n = 55), 4.7% (n = 17) and 80.1% (n = 289), respectively, showing a clear preference for adoption.

Of women with a history of examination or treatment of infertility (n = 374), 36.6%, 11.2% and 52.1% favored UTx, gestational surrogacy and adoption, respectively, whereas these respective rates were 28.4%, 7.4% and 64.1% for women without a history of examination or treatment of infertility (n = 2173). Thus, those who had undergone an examination or treatment were more likely to favor UTx and gestational surrogacy, and less likely to favor adoption (p<0.001). Women with a history of advanced ART, including IVF treatment (n = 94) showed particularly high support for UTx, with rates of 55.3% (n = 52), 9.6% (n = 9) and 36.2% (n = 34) for UTx, gestational surrogacy and adoption, respectively.

A total of 89.9% (n = 2786) of the respondents did not support at least one of UTx, gestational surrogacy and adoption (Q19, multiple answers allowed). The rates of opposition to UTx, gestational surrogacy and adoption were 21.9%, 63.3% and 7.0%, respectively. The rates for the option viewed least favorably for UTx, gestational surrogacy and adoption (Q20) were 22.2%, 69.5% and 8.5%, respectively.

### Social acceptance

Regarding social and ethical acceptance for UTx (Q21), the rates were 15.7% for "UTx should be permitted", 77.6% for "UTx should be permitted with further discussion", and 6.7% for "UTx should not be permitted, even with discussion". The percentages of respondents who gave the third answer in the 25–29, 30–34 and 35–39 age groups were 20.7%, 33.2% and 46.2%, respectively, showing an increasing tendency with age. There were no effects of marriage, children or residential area (p = 0.185, 0.361, 0.628, respectively). A significantly higher percentage of women with a history of examination or treatment of infertility indicated that UTx should be permitted compared to women without this history (20.2%, 90/445 vs. 15.0%, 397/2653, p = 0.002). Among women with a history of IVF treatment, 45.0% (n = 49) stated that UTx should be permitted and only 2.8% (n = 3) felt that UTx should not be permitted. Also, women who were aware that UTx was used overseas (Q3) were significantly more likely to indicate that UTx should be permitted (p = 0.026).

### Personal opinion

For personal opinion on UTx (Q22), the rates were "Favor very much" 4.8%, "In favor" 39.4%, "No opinion" 47.5%, "Against" 7.4%, and "Absolutely against" 0.9%. "No opinion" was most common, but support ("In favor" + "Favor very much", 44.2%, n = 1369) was higher than opposition ("Against" + "Absolutely against", 8.3%, n = 257).

The rates of support for UTx in the 25–29, 30–34 and 35–39 age groups were 35.6% (n = 487), 34.7% (n = 475) and 29.7% (n = 407), respectively, and those in opposition were 24.7% (n = 62), 36.6% (n = 94) and 39.4% (n = 101), respectively, showing tendencies for more support in the younger group and more opposition in the older group. Among women with children, 47.7% (n = 608) were supportive of UTx and 6.8% (n = 86) were in opposition, whereas these rates for women without children were 41.7% (n = 761) and 9.4% (n = 86), respectively, showing tendencies for more support in women with children. In regression analysis of age, marriage, children, and residential area, age and children had a significant relationship with support for UTx (both p<0.001).

Of women (n = 445) with a history of examination or treatment of infertility, 52.4% (n = 233) supported UTx and 5.8% (n = 26) opposed, whereas these rates in women (n = 2653) without this history were 42.8% (n = 1136) and 8.7% (n = 231), respectively, showing that women with a history of examination or treatment of infertility were significantly more likely to support UTx (p<0.001). Women (n = 109) with a history of IVF treatment had rates of 68.8% (n = 75) and 0% (n = 0) for support and opposition, respectively, and thus were particularly supportive of UTx. For women (n = 466) who thought they did not want a child, the rate of opposition to UTx was significantly higher than that for all women (16.1% vs. 8.3%, p<0.001).

Of women (n = 545) who were aware of performance of UTx in humans overseas, 51.7% (n = 282) supported UTx and 5.5% (n = 30) were opposed. These women were significantly more supportive of UTx than women who were unaware of overseas UTx, in whom the respective rates were 42.6% (n = 1087) and 8.9% (n = 227) (p<0.001). Of women who chose gestational surrogacy (n = 203) and adoption (n = 1589) as alternative methods to UTx in Q18, 45.3% (n = 92) and 36.9% (n = 586) supported UTx, and 4.4% (n = 9) and 10.9% (n = 174) were opposed UTx, respectively. Thus, even women who preferred gestational surrogacy or adoption were generally in favor of UTx.

## Discussion

The successful delivery after UTx described by Brännström et al. [4] has provided great hope to women with UFI. This has led to more discussion of clinical application of UTx in Japan. UTx is viewed as an organ transplant to improve quality of life, in contrast to transplant of a vital organ. Therefore, discussion of the bioethics of UTx is needed as a medical intervention in the origin of life. Alternatives include gestational surrogacy and adoption, and the choice among these methods depends on personal opinions and social and religious background. There has been no large-scale survey of UTx in Japan, and thus the current results are important for understanding the attitude of Japanese women of reproductive age toward UTx.

Our survey had several features to ensure collection of accurate and effective data without bias. Although the subjects were all of reproductive age (25 to 39 years), we hypothesized that attitudes toward UTx would still depend on age, and thus data were analyzed in 5-year age groups with about equal numbers of respondents as the primary endpoint. Each age group included percentages of unmarried women and residential areas corresponding to the Japanese population census. Inappropriate responses, which are always present in Internet surveys, were excluded and the orders of options in several questions were randomized to prevent order bias. Objective information was provided to minimize information bias on UTx. The characteristics of the respondents showed that the population sampling was performed as planned. In all age groups, <20% of respondents were aware of implementation of UTx overseas, which suggests that UTx is not well known in Japan.

For candidate donors and recipients (Q8-11), brain-dead/non-heart-beating donors were favored, which indicates consideration of the burden on UTx donors, followed by mothers. For recipients, those with uterine agenesis were favored. Recipients after hysterectomy due to postpartum hemorrhage were favored least, but this opinion depended on the background that a woman had at least one child. Based on these results, transplant from brain-dead/non-heart-beating donors to recipients with uterine agenesis (e.g. Mayer-Rokitansky-Küster-Hauser syndrome) is most likely to be accepted by the public. UTx between a living donor and recipient was supported for transplant from a mother to a recipient with uterine agenesis.

With regard to personal opinion of UTx (Q12), about half of the respondents had "No opinion", which suggests that UTx is not fully understood by the public. In a situation in which women were asked to imagine that they had UFI, more than a half who chose the answer "I do not want UTx" also had no wish to have a child; therefore, this increased the rate of respondents with no desire for UTx. Many respondents who desired UTx already had children and many also had a history of examination or treatment of infertility, which suggests that their wish for UTx depended on feelings of well-being and satisfaction of having children and a desire for treatment of infertility. Regarding reasons for wanting or not wanting UTx (Q13-16), the answer "We want a child with genes inherited from us" was the highest, suggesting that an advantage of UTx over adoption is the ability to have a child with genes inherited from parents. The reasons for not wanting UTx were mainly the burdens placed on UTx donors and recipients, rather than effects on children.

In comparison of UTx with alternative treatment (Q17-20), adoption was most supported and gestational surrogacy received less support. These findings reflect the current system in Japan, in which adoption is only available, while gestational surrogacy is forbidden [9,16–18]. Surprisingly, UTx was considerably supported more than gestational surrogacy. These results may be related to news in the same year as that of the survey on delivery after UTx [4], about an Australian couple denied the right to take a child born to a Thai woman as a surrogate mother [19], and an incident in which Japanese persons asked many women to assist with gestational surrogacy [20]. There were no differences in opinions based on age and residential area; however, the percentage of married women with children in favor of UTx was higher than that in favor of adoption, which suggests that the potential to have a child with genes inherited from parents is likely to increase support for UTx. Women who did not want a child may have supported adoption because they did not expect a positive outcome from ART and they felt a burden. Women with a history of examination or treatment of infertility were more likely to support UTx and gestational surrogacy than those without this history, with this being particularly true for women with a history of IVF treatment, suggesting a tendency to use advanced medical technology to assist with having a child.

Regarding social acceptance (Q21), many respondents were positive, and most women with a history of IVF treatment were in favor, suggesting positive views of UTx. Women who were aware of the use of UTx overseas were proactively in favor, which indicates a social effect of the overseas studies on Japanese society. Women in the 35–39 age group were more negative compared with women in the 25–29 age group, indicating a more conservative approach in the older age group.

Personal opinions on UTx (Q22) were positive for many respondents, and particularly among younger women, those who had children, those with a history of examination or treatment of infertility, and those who were aware of use of UTx overseas. However, similarly to Q12, the common answer was "No opinion", which indicated that many respondents did not fully understand UTx and that information on UTx in humans is still insufficient. Respondents who preferred gestational surrogacy or adoption also had positive opinions on UTx, suggesting a desire to allow women with UFI to have a child.

The limitations of this survey include selection bias, in that the respondents may have been interested in UTx to some extent, even though they were collected randomly; and information bias, which may not have been prevented despite the objective explanation given in the survey. Thus, respondents might have been more likely to support UTx. Furthermore, successful delivery after UTx overseas and negative incidents in gestational surrogacy were reported in the same year as that of the survey, and this may have caused UTx to be viewed more positively than gestational surrogacy. In addition, the subjects in this study were women of reproductive age. The attitude of Japanese men toward UTx probably differs from that of women, and further studies are required in men. The effectiveness of UTx remains uncertain and questionnaires in this area require that respondents are given a thorough explanation, including complications for donors and recipients. However, questionnaires conducted via the Internet may have more inappropriate answers and decreased quality of data, with results obtained from respondents with limited information. These result may differ from those from a study in respondents who received a full explanation. This may be particularly true for the comparison between UTx and gestational surrogacy, since the characteristics and detailed risks of these procedures were not fully explained to the respondents in this study and opinions on these issues may be changed by this explanation.

This study is specific to the social context in Japan and attitudes toward new ART depend on social, religious and political background. In particular, regulations of reproductive technology via a third party and social acceptance differ considerably among countries [18,21–26]. The general public in countries in which gestational surrogacy or adoption is permitted may have less demand for UTx in comparison with countries in which alternatives to gestational surogacy are not permitted. Therefore, further studies are required in Japan and in other countries to evaluate attitudes toward UTx, and it would be preferable to have a global discussion on UTx as a potential new ART.

## Conclusion

We investigated opinions on UTx of women of reproductive age in Japan and compare the attitude by age as the primary endpoint. Many Japanese women of reproductive age felt that UTx is socially and personally acceptable, with this view particularly indicated by younger women and women with a history of treatment of infertility. However, concerns remain among these women that require further discussion. Many responders felt that the best donors were brain-dead/non-heart-beating donors and mothers, and that the best recipients were patients with uterine agenesis. The major reason for support for UTx was the desire for a child to have genes inherited from parents. On the contrary, the main reason for not desiring UTx was that they did not want to place themselves and others at risk, which suggests that burdens on donors and recipients were considered to be more concrete than risks for children. The priority order of options for women with UFI to have a child was adoption, UTx, and gestational surrogacy, with UTx significantly favored in comparison with gestational surrogacy. However, the answer "No opinion" was most common regarding attitude toward UTx, which indicates that UTx is not fully understood in Japan. Therefore, provision of more information and public discussion is required, after which it will be necessary to evaluate if clinical application of UTx should be permitted in Japan.

## Supporting Information

S1 Appendix Appendix A: Internet questionnaire on UTx.
(DOCX)

S2 Appendix Appendix B: Explanation of UTx and questions for confirmation of understanding.
(DOCX)

## References

[1] Arora KS, Blake V. Uterus transplantation: the ethics of moving the womb. Obstet Gynecol 2015;125:971–974. 10.1097/AOG.0000000000000707 25751213

[2] Johannesson L, Dahm-Kähler P, Eklind S, Brännström M. The future of human uterus transplantation. Womens Health (Lond Engl) 2014;10:455–467. 25259905 10.2217/whe.14.22

[3] Kisu I, Banno K, Mihara M, Suganuma N, Aoki D. Current status of uterus transplantation in primates and issues for clinical application. Fertil Steril 2013;100:280–94. 10.1016/j.fertnstert.2013.03.004 23557761

[4] Brännström M, Johannesson L, Bokström H, Kvarnström N, Mölne J, Dahm-Kähler P, et al Livebirth after uterus transplantation. Lancet 2015;385:607–616. 10.1016/S0140-6736(14)61728-1 25301505

[5] 2012 Report of the Ethics Committee and Registration and Investigation Subcommittee of the Japan Society of Obstetrics and Gynecology. Acta Obstetrica et Gynaecologica Japonica 2014;66: 2445–2454.(Japanese)

[6] Chambers GM, Sullivan EA, Ishihara O, Chapman MG, Adamson GD. The economic impact of assisted reproductive technology: a review of selected developed countries. Fertil Steril 2009;91:2281–2294. 10.1016/j.fertnstert.2009.04.029 19481642

[7] Semba Y, Chang C, Hong H, Kamisato A, Kokado M, Muto K. Surrogacy: donor conception regulation in Japan. Bioethics 2010;24:348–357. 10.1111/j.1467-8519.2009.01780.x 20002072

[8] Brinsden PR. Gestational surrogacy. Hum Reprod Update 2003;9:483–491. 14640380 10.1093/humupd/dmg033

[9] Kisu I, Banno K, Mihara M, Iida T, Yoshimura Y. Current status of surrogacy in Japan and uterine transplantation research. Eur J Obstet Gynecol Reprod Biol 2011;158:135–140. 10.1016/j.ejogrb.2011.04.037 21632170

[10] Goldfarb KE. Developmental logics: Brain science, child welfare, and the ethics of engagement in Japan. Soc Sci Med 2015; 143:271–278. 10.1016/j.socscimed.2014.11.036 25530189

[11] Hibino Y, Shimazono Y, Kambayashi Y, Hitomi Y, Nakamura H. Attitudes towards cross-border reproductive care among infertile Japanese patients. Environ Health Prev Med 2013;18:477–484. 10.1007/s12199-013-0345-7 23749591 PMC3824726

[12] Ozkan O, Akar ME, Ozkan O, Erdogan O, Hadimioglu N, Yilmaz M, et al Preliminary results of the first human uterus transplantation from a multiorgan donor. Fertil Steril 2013;99:470–476. 10.1016/j.fertnstert.2012.09.035 23084266

[13] Erman Akar M, Ozkan O, Aydinuraz B, Dirican K, Cincik M, Mendilcioglu I, et al Clinical pregnancy after uterus transplantation. Fertil Steril 2013;100:1358–1363. 10.1016/j.fertnstert.2013.06.027 23830110

[14] Johannesson L, Kvarnström N, Mölne J, Dahm-Kähler P, Enskog A, Diaz-Garcia C, et al Uterus transplantation trial: 1-year outcome. Fertil Steril 2015;103:199–204. 10.1016/j.fertnstert.2014.09.024 25439846

[15] Brännström M, Johannesson L, Dahm-Kähler P, Enskog A, Mölne J, Kvarnström N, et al First clinical uterus transplantation trial: a six-month report. Fertil Steril 2014; 101:1228–1236. 10.1016/j.fertnstert.2014.02.024 24582522

[16] Shirai Y. Japanese attitudes toward assisted procreation. J Law Med Ethics 1993;21:43–53. 11652121 10.1111/j.1748-720x.1993.tb01229.x

[17] Suzuki K, Hoshi K, Minai J, Yanaihara T, Takeda Y, Yamagata Z. Analysis of national representative opinion surveys concerning gestational surrogacy in Japan. Eur J Obstet Gynecol Reprod Biol 2006:126:39–47. 16171926 10.1016/j.ejogrb.2005.07.030

[18] Saito Y, Matsuo H. Survey of Japanese infertile couples’ attitudes toward surrogacy. J Psychosom Obstet Gynaecol 2009;30:156–161. 10.1080/01674820802429435 19591054

[19] Fears of surrogacy ban after Australian couple deserts Thai surrogate mother of baby with Down syndrome. ABC News. 2 Aug 2014. Available at: External link http://www.abc.net.au/news/2014-08-02/outrage-as-australian-parents-desert-surrogate-mother/5643074 Accessed Aug 29, 2015.

[20] Japanese man fathers 16th baby via surrogate in Thailand. ABC News. 10 Sep 2014.Available at: External link http://www.abc.net.au/news/2014-09-10/japanese-surrogacy-man-has-another-baby/5732856 Accessed Aug 29, 2015.

[21] Schenker JG. Assisted reproduction practice in Europe: legal and ethical aspects. Hum Reprod Update 1997;3:173–184. 9286740 10.1093/humupd/3.2.173

[22] Poote AE, van den Akker OB. British women's attitudes to surrogacy. Hum Reprod. 2009;24:139–145. 10.1093/humrep/den338 18794160

[23] Daniluk JC, Koert E. Childless Canadian men's and women's childbearing intentions, attitudes towards and willingness to use assisted human reproduction. Hum Reprod 2012;27:2405–2412. 10.1093/humrep/des190 22684907

[24] Constantinidis D, Cook R. Australian perspectives on surrogacy: the influence of cognitions, psychological and demographic characteristics. Hum Reprod 2012;27:1080–1087. 10.1093/humrep/der470 22294557

[25] Kian EM, Riazi H, Bashirian S. Attitudes of Iranian infertile couples toward surrogacy. J Hum Reprod Sci. 2014; 7:47–51. 10.4103/0974-1208.130847 24829531 PMC4018798

[26] Baykal B, Korkmaz C, Ceyhan ST, Goktolga U, Baser I. Opinions of infertile Turkish women on gamete donation and gestational surrogacy. Fertil Steril 2008;89:817–822. 10.1016/j.fertnstert.2007.04.022 18406837

