# Supplementary material for: Survey of Attitudes toward Uterus Transplantation among Japanese Women of Reproductive Age: A Cross-Sectional Study
Source: PLoS One. 2016 May 20;11(5):e0156179. doi: 10.1371/journal.pone.0156179 (PMC4874691; doi:10.1371/journal.pone.0156179)
Supplement: S1 Appendix — Appendix A: Internet questionnaire on UTx. (DOCX) [file pone.0156179.s001.docx]

**S1 Appendix.**

**Appendix A : Internet questionnaire on UTx**

Q1: Do you have children? If yes, circle the number of children. If you do not have children, answer if you would or would not like to have children.

1. 1 child 4. I currently want to have children

2. 2 children 5. I want to have children in the future

3. 3 children or more 6. I don't want to have children

7. I am currently pregnant

Q2: Have you undergone an examination or treatment for infertility? Choose answers below (Check all that apply)

1. Infertility examination 4. IVF treatment

2. Timing therapy 5. Others

3. Artificial insemination 6. I have not undergone treatment for infertility

Q3: Have you heard that UTx was recently performed in Sweden and Turkey?

1. Yes

2. No

Q4: What is your response when you hear the term 'UTx' ?

1. Very interested in UTx. 4. No interest in UTx.

2. Interested in UTx. 5. I don't understand UTx well.

3. Less interested in UTx.

Explanation of UTx and questions for confirmation of understanding (Appendix B)

Q5-7: Questions to confirm understanding of UTx

Q8: Who is appropriate as a donor in UTx? (Check all that apply)

1. Mother

2. Sister

3. Relative excluding mother and sister

4. Friend or acquaintance

5. Anonymous third-party

6. Person with gender identity disorder (female to male)

7. Brain-dead/non-heart-beating donor

Q9: If you chose several items in the above question, please indicate your top choice.

Q10: Who is appropriate as a recipient in UTx? (Check all that apply)

1. Woman who has no uterus due to a congenital disease.

2. Woman who lost her uterus due to a malignant uterine tumor.

3. Woman who lost her uterus due to a benign uterine tumor.

4. Woman whose uterus was removed as a life-saving procedure after postpartum hemorrhage

5. Woman whose uterus has intrauterine synechiae (Asherman's syndrome)

Q11: If you chose several items in the above question, please indicate your top choice.

Q12: If you lost your uterus or had no uterus, would you want to have a child via UTx?

1. Yes. 2. No opinion 3. No.

Q13: What are your reasons for wanting UTx? (Check all that apply)

(If you chose answer 3 for Q12, do not answer this question)

1. We want a child with genes inherited from us.

2. There are no ethical problems because the sperm and ovum are ours, even though the uterus is not.

3. If the uterus is removed after delivery, immunosuppressants are transiently administered.

4. Maternal feelings are improved by self-pregnancy and delivery, in contrast to gestational surrogacy and adoption.

5. Women suffering from uterine factor infertility will have the hope to have a child.

6. We want a child by self-delivery who is recognized as ours (the couple's) in the Japanese Civil Code.

7. Other reasons

Q14: If you chose several items in the above question, please indicate your top choice.

Q15: What are your reasons for not wanting UTx? (Check all that apply)

(If you chose answer 1 for Q12, do not answer this question)

1. Transplant should be performed only for vital organs, and not for having a child.

2. I am afraid of the surgery itself.

3. I am worried about the effect of immunosuppressants on a child (anomaly).

4. Immunosuppressants are needed.

5. I do not want pregnancy and delivery to be risks for myself and the donor.

6. Hope should not be easily given to women who have lost their uterus.

7. Other reasons

Q16: If you chose several items in the above question, please indicate your top choice.

Q17: Which of UTx, gestational surrogacy and adoption do you support?

(Check all that apply)

1. UTx 2. Gestational surrogacy 3. Adoption 4. None

Q18: If you chose several items in the above question, please indicate your top choice.

(If you chose answer 4 on Q17, do not answer this question)

Q19: Which of UTx, gestational surrogacy and adoption do not you support?

(Check all that apply)

1. UTx 2. Gestational surrogacy 3. Adoption 4. In favor of all

Q20: Which method do you least support?

(If you chose answer 4 on Q19, do not answer this question)

Q21: Do you think that UTx should be ethically permitted by the public?

1. UTx should be permitted.

2. Challenges remain, but UTx should be permitted after further discussion

3. UTx should not be permitted, even with further discussion.

Q22: What do you think about UTx?

1. Very much in favor

2. In favor

3. No opinion

4. Against

5. Absolutely against
