# Supplementary material for: Survey of Attitudes toward Uterus Transplantation among Japanese Women of Reproductive Age: A Cross-Sectional Study
Source: PLoS One. 2016 May 20;11(5):e0156179. doi: 10.1371/journal.pone.0156179 (PMC4874691; doi:10.1371/journal.pone.0156179)
Supplement: S2 Appendix — Appendix B: Explanation of UTx and questions for confirmation of understanding. (DOCX) [file pone.0156179.s002.docx]

**S2 Appendix.**

**Appendix B: Explanation of UTx and questions for confirmation of understanding**

**< Introduction >**

In Japan, one out of 6 couples suffers from "infertility", i.e., they want a child, but do not have one. Many infertile patients can be treated due to medical developments. However, some women have problems with the uterus itself, and consequently they cannot become pregnant or deliver a child. This may be because they have no uterus naturally or they have lost the uterus due to surgery for a uterine tumor. New assisted reproductive technology, which is referred to as uterus transplantation (UTx), may allow these women to have a child.

**< What is UTx? >**

In UTx, the recipient obtains a uterus from a donor, becomes pregnant, and delivers a child. A fertilized egg is derived from the sperm and ovum of the couple; therefore, the newborn is genetically related to the parents.

Other options to have a child include gestational surrogacy and adoption. However, gestational surrogacy is forbidden in Japan due to ethical problems, and the Japanese Civil Code does not recognize a child born from a surrogate mother as the child of the parents who made a fertilized egg with their sperm and ovum.


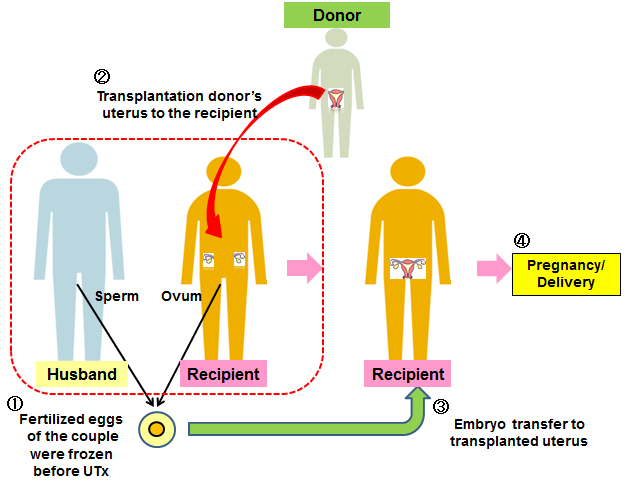


**Explanation:**
**A fertilized egg of the couple is cryopreserved and the donor provides the uterus.** **The embryo is transfered into the transplanted uterus from the donor for pregnancy and delivery.**

UTx has the advantage that a woman can carry a fetus in her own uterus and deliver it by herself; however, she must undergo transplantation and transiently take immunosuppressants after surgery until delivery to prevent rejection of the transplanted organ as a non-self organ.

**< Current status of uterus transplantation >**

Challenges for UTx include use of immunosuppressants, surgical safety, effects on the fetus, and donor burdens. UTx is ongoing in Sweden and Turkey as clinical research (experimental level). The Swedish team has achieved delivery in 3 women who underwent UTx.

**Questions to confirm understanding of UTx**

Q5: What is the person providing an organ for organ transplant known as?

1) Donor 　　　　2) Recipient　　　 3) Patient with uterine factor infertility

Q6: What drugs are administered to a patient who underwent UTx to prevent rejection?

1) Antibiotics 2) Analgesics 3) Immunosuppressants

Q7: Which of the following statements is wrong?

1) A newborn born by UTx is genetically related to the couple who provided the sperm and ovum.

2) UTx is a technique developed for women who cannot become pregnant or deliver a child due to uterine problems.

3) Clinical research on UTx has started in foreign countries.

4) In UTx, there is no concern about immunosuppressants or surgical risks.
